# Ultrasound Image Features under Deep Learning in Breast Conservation Surgery for Breast Cancer

**DOI:** 10.1155/2021/6318936

**Published:** 2021-09-17

**Authors:** Hongxu Zhang, Haiwang Liu, Lihui Ma, Jianping Liu, Dawei Hu

**Affiliations:** ^1^Department of Breast Surgery, Affiliated Hospital of Chengde Medical University, Chengde 067000, Hebei, China; ^2^Department of Pathology, Affiliated Hospital of Chengde Medical university, Chengde 067000, Hebei, China

## Abstract

This study was to analyze the effect of the combined application of deep learning technology and ultrasound imaging on the effect of breast-conserving surgery for breast cancer. A deep label distribution learning (LDL) model was designed, and the semiautomatic segmentation algorithm based on the region growing and active contour technology (RA) and the segmentation model based on optimized nearest neighbors (ON) were introduced for comparison. The designed algorithm was applied to the breast-conserving surgery of breast cancer patients. According to the difference in intraoperative guidance methods, 102 female patients with early breast cancer were divided into three groups: 34 cases in W1 group (ultrasound guidance based on deep learning segmentation model), 34 cases in W2 group (ultrasound guidance), and 34 cases in W3 group (palpation guidance). The results revealed that the tumor area segmented by the LDL algorithm constructed in this study was closer to the real tumor area; the segmentation accuracy (AC), Jaccard, and true-positive (TP) values of the LDL algorithm were obviously greater than those of the RA and ON algorithms, while the false-positive (FP) value was significantly lower in contrast to the RA and ON algorithms, showing statistically observable differences (*P* < 0.05); the actual resection volume of the patients in the W1 group was the closest to the ideal resection volume, which was much smaller in contrast to that of the patients in the W2 and W3 groups, showing statistical differences (*P* < 0.05); the positive margins of the patients in the W1 group were statistically lower than those in the W2 and W3 groups (*P* < 0.05). In addition, 1 patient in the W1 group was not satisfied with the cosmetic effect, 3 patients in the W2 group were not satisfied with the cosmetic effect, and 9 patients in the W3 group were not satisfied with the cosmetic effect. Finally, it was found that the ultrasound image based on the deep LDL model effectively improved the AC of tumor resection and negative margins, reduced the probability of normal tissue being removed, and improved the postoperative cosmetic effect of breast.

## 1. Introduction

Breast cancer is a malignant tumor that occurs on the ductal epithelium and terminal ductal epithelium of the breast. It can manifest as breast lumps, nipple discharge, nipple retraction, skin adhesions, skin edema, and breast pain [1]. The invasive breast cancer is still major diseases threatening the lives and health of women [2]. In the past, the breast cancer was treated by removing the breasts for the classic surgical treatment, the expanded radical mastectomy, and modified radical mastectomy, which would leave women's chest long and ugly scar after the surgery [3]. In addition, large-scale mastectomy and axillary lymph node dissection will inevitably bring more surgical complications, such as the formation of postoperative scar tissue, which limits the range of movement of the upper limbs; the lymphatic circulation of the upper limbs is blocked, which leads to swelling of the upper limbs [4]. Therefore, modern clinical treatment recommends breast cancer conserving surgery. Compared with radical mastectomy, breast-conserving surgery has the characteristics of less trauma and less pain. While preserving the integrity of the breast shape, it also takes into account postoperative functional recovery, so the curative effect can be comparable to radical mastectomy by combining with postoperative comprehensive treatment [5, 6]. Although the current surgery is still generally based on modified radical mastectomy, breast-conserving surgery will gradually replace modified radical mastectomy as the main surgical procedure with the development of early diagnosis technology.

At present, the auxiliary methods suitable for breast-conserving surgery for breast cancer include intraoperative ultrasound guidance and palpation guidance. Although palpation guidance is a clinical standard surgical procedure, its performance in assessing the extent of resection of the disease is low, and excessive resection scope and positive margins may occur, which will not only affect the surgical effect but also lead to low cosmetic effect and unsuccessful surgery [7]. Ultrasound, as an imaging method widely used in clinical purposes, has developed from the initial diagnosis of breast cancer to aid surgical treatment. It can play an important role in breast cancer conserving surgery, and clinical ultrasound-guided breast-conserving surgery is necessary [8]. Deep learning, as a new field in machine learning research, allows computers to simulate the mechanism of the human brain to solve problems and enables the computers to improve their ability to solve problems on their own without human supervision [9, 10]. Clinical application of deep learning technology to ultrasound image segmentation is also a major trend. For example, the shape, color, and texture of the lesion can be classified through accurately segmenting the lesion, so that the machine can complete the doctor's diagnosis process or visualizing the images in three dimensions can effectively improve the diagnostic performance of doctors [11]. A multilabel classification task refers to a piece of data that may have one or more labels. For example, a patient's physical examination report may be labeled with multiple labels, such as high blood pressure and high blood sugar. Multilabel learning algorithms can be divided into problem conversion methods and algorithm adaptation methods. The problem conversion method is to convert the multilabel classification into other mature scenarios, so that the data can adapt to the algorithm, and the algorithm adaptation method is to adapt popular learning techniques to deal with multilabel data and let the algorithm adapt to the data. Therefore, it is necessary to combine the deep learning and ultrasound.

In short, the combined application of deep learning technology and imaging technology can be well developed in the field of medical diagnosis and treatment. In this study, a deep LDL model was designed and applied to breast cancer patients during breast-conserving surgery. The combined application of deep learning technology and ultrasound imaging on the effect of breast-conserving surgery for breast cancer was comprehensively evaluated by analyzing the amount of tumor resection, margins, and postoperative cosmetic effects in breast cancer patients.

## 2. Materials and Methods

### 2.1. Research Objects

One hundred and two female patients with early breast cancer (the clinical TNM staging is stage I and II) admitted in the hospital from February 10, 2018, to February 10, 2021, were selected as the research subjects. The patients with multicenter lesions, preoperative neoadjuvants, breast cancer surgery history, nontactile breast cancer, and invasive breast cancer were excluded. According to the different guidance methods used in the surgery, the research objects were rolled into three groups: 34 cases in W1 group (ultrasound guidance based on deep learning segmentation model), 34 cases in W2 group (ultrasound guidance), and 34 cases in W3 group (palpation guidance). This study had been approved by the Medical Ethics Committee of hospital, and the patients and their families had known the situation of the study and signed the informed consent forms.

### 2.2. Guidance Methods

Ultrasound guidance was performed as follows: Before the surgery, SonoScape was used to open the M11 ultrasound color Doppler diagnostic instrument to mark the tumor boundary on the patient's skin surface, and set 1 cm around the tumor as the ideal resection margin. After anesthesia, an appropriate incision was made, and an ultrasound probe was adopted to guide the surgical resection in real time. During the process, it had to pay attention to the gentle operation to avoid tumor compression. Ultrasonic testing was performed on the isolated specimens after surgery to ensure that the tumor was cleanly removed.

Palpation guidance was performed as follows. The SonoScape was adopted to open the M11 ultrasound color Doppler diagnostic instrument to mark the tumor boundary on the skin surface of the patient before the surgery, and set 1 cm around the tumor as the ideal resection margin. After anesthesia, a suitable incision was cut on the skin, and the palpation guidance was realized by figures. The tumor was marked and then completely removed based on past experience.

### 2.3. Pathological Examination and Data Collection

The pathological examination was performed on the tumor tissue resected by the two guiding methods; the circumferences (length, width, and height) of the tumor and the resected tissue were measured and the distance between each resection edge and the tumor edge was measured (obtain the longest/shortest resection edge). The resection edge was observed to check if the tumor cells were visible. Under a microscope, if there was no ink staining on the tumor tissue, it can be judged as a negative margin.

The tumor volume (V1), ideal resection tissue volume (V2), and actual resection tissue volume (V3) were calculated with following equations:

(1) $$\begin{array}{c} V1=\frac{4}{3}\pi •\frac{\alpha }{2}•\frac{\beta }{2}•\frac{\lambda }{2}, \end{array}$$

(2) $$\begin{array}{c} V2=\frac{4}{3}\pi •\left(\frac{\alpha }{2}+1\right)•\left(\frac{\beta }{2}+1\right)•\left(\frac{\lambda }{2}+1\right), \end{array}$$

(3) $$\begin{array}{c} V3=\frac{4}{3}\pi •\frac{\alpha *}{2}•\frac{\beta *}{2}•\frac{\lambda *}{2}. \end{array}$$

In (1)–(3), *α*, *β*, and *λ* referred to the length, width, and height of the tumor, respectively, and *α*^*∗*^, *β*^*∗*^, and *λ*^*∗*^ represented the length, width, and height of the actual resection tissue, respectively.

The age, weight, height, and tumor location of the patients were collected before the surgery. Long-term follow-up was carried out after the surgery. The breast shape, incision healing, and nipple, and areola positions of the patients were observed 2 and 5 months after the surgery.

### 2.4. Deep LDL Model

**Figure 1:**
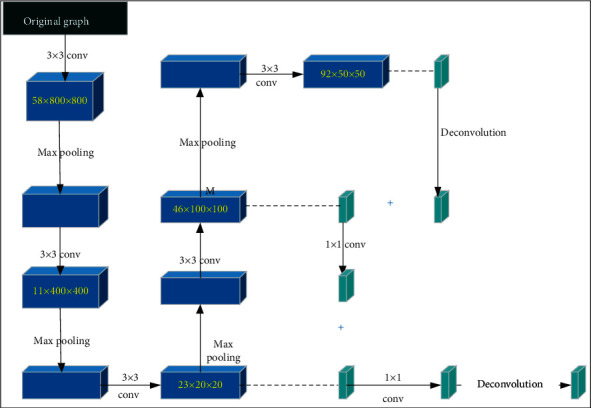
The architecture of LDL.

LDL can label an instance very naturally and assign a performance value to each label of the instance as much as possible. Compared with the traditional single-label learning, it showed more degrees of freedom to obtain more effective information [12]. Therefore, an encoder-decoder architecture deep network was designed based on LDL (Figure 1), which was composed of input layer, convolution module, pooling layer, subsequent branches of the convolution module, and softmax layer.

The network can perform Softmax operation [13] to obtain the label distribution map required. When the classification probability and cross entropy loss were calculated, the equations could be written as follows:

(4) $$\begin{array}{c} P_{i}=\frac{a^{i}}{\sum_{i}a^{i}}, \end{array}$$

(5) $$\begin{array}{c} \text{Loss}=-\sum_{m}y_{m}\log\;P_{m}. \end{array}$$

In (4) and (5), *P* was the classification probability, *a*_*i*_ was the element contained in the input data, and *y*={*y*_0_, *y*_1_, *y*_2_,…, *y*_*n*_} ∈ {0,1} represented the category. There were two categories in this study: tumor and background. When it was the tumor category, Loss(*P*, *y*)=−log *P*_1_ could be met; when it was the background category, Loss(*P*, *y*)=−log *P*_0_ was satisfied. From this, the fuzzy label distribution map can be initially obtained.

A label distribution fitting term [14] was proposed in this study to further integrate the obtained fuzzy label distribution map into the LDL model; then, the new energy function could be expressed as follows:

(6) $$\begin{array}{c} H=\varepsilon +\phi +D. \end{array}$$

In equation (6), *ε* represented the gray-scale fitting term; *ϕ* and *D* represented the label fitting term and the regularization term, respectively. The following equation could be obtained by introducing the bias field hypothesis theory:

(7) $$\begin{array}{c} \varepsilon =\int_{\Phi }{\left(M\left(x,y\right)-C\left(x,y\right)N\left(x,y\right)\right)}^{2}\text{d}x\text{d}y. \end{array}$$

In the above equation, *M* represented the image to be observed, *N* represented the real image, *C* was the bias field, and *Փ* referred to the interval. Then, the concept of local clustering properties was introduced. It was assumed that there was a circle with *r* as radius and *s* as center, the paranoid field value of any point *s*^*∗*^ in this circle was similar to the center *s*. In addition, the values of the real image on multiple intervals [Փ1, Փ2, ... Փ*n*] were [*v*1, *v*2,... *v*_*n*_], respectively; then, equation (7) could be evolved as follows:

(8) $$\begin{array}{c} \varepsilon =\int \left(\overset{n}{\underset{i=1}{\sum }}\int L\left(s-s*\right){\left(M\left(s*\right)-C\left(s\right)v_{i}\right)}^{2}\right)\text{d}s. \end{array}$$

A label distribution fitting item was also added, which was different from the traditional model:

(9) $$\begin{array}{c} \phi =\overset{n}{\underset{i=1}{\sum }}\int_{\Phi }{\left(S\left(s*\right)-v_{i}\right)}^{2}\text{d}s. \end{array}$$

In equation (9), *S* (*s*^*∗*^) was the pixel value obtained from the label learning map and represented the tumor probability value in this study. Adding the abovementioned label distribution fitting item can solve the different qualitative gray levels. Therefore, the final energy function can be expressed as:

(10) $$\begin{array}{c} H=\int \left(\overset{n}{\underset{i=1}{\sum }}\int L\left(s-s*\right){\left(M\left(s*\right)-C\left(s\right)v_{i}\right)}^{2}\right)\text{d}s+\overset{n}{\underset{i=1}{\sum }}\int_{\Phi }{\left(S\left(s*\right)-v_{i}\right)}^{2}\text{d}s+\left|D\right|. \end{array}$$

### 2.5. Evaluation Indicators of Segmentation Performance

The semiautomatic segmentation algorithm based on the region growing and active contour technology (RA) and the segmentation model based on optimized nearest neighbors (ON) were introduced for comparison to further analyze the performance of the designed model. The accuracy (AC), true positive (TP), false positive (FP), and Jaccard were selected as the evaluation indicators, which could be expressed as follows:

(11) $$\begin{array}{c} \text{AC}=\frac{\left|T_{1}\cap T_{2}\right|\cup \left|C_{1}\cap C_{2}\right|}{T_{1}\cap C_{1}},\\ \text{TP}=\frac{\left|T_{1}\cap T_{2}\right|}{T_{1}},\\ \text{FP}=\frac{\left|T_{1}\cap T_{2}-T_{1}\right|}{\left|T_{1}\right|},\\ \text{Jaccard}=\frac{\left|T_{1}\cap T_{2}\right|}{\left|T_{1}\cup T_{2}\right|}. \end{array}$$

In the above equations, *T*_1_ referred the tumor area obtained by manual segmentation, and *T*_2_ represented the tumor area obtained by the model segmentation.

### 2.6. Statistical Analysis

The data of this study was analyzed by SPSS19.0 version statistical software; the measurement data were expressed by the mean ± standard deviation (‾*x* ± *s*), and the count data were expressed in the form of percentage (%). One-way analysis of variance was used for pairwise comparison. The difference was statistically significant at *P* < 0.05.

## 3. Results

### 3.1. Image Segmentation Performance of Deep LDL Model

**Figure 2:**
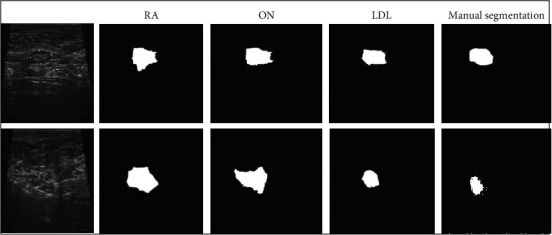
Comparison on image segmentation effects of different algorithms. (a, b) Original images.

Figure 2 shows the image segmentation effects of the three algorithms on some samples. It revealed that the tumor area segmented by the LDL algorithm constructed was closer to the real tumor area. Although the RA algorithm and the ON algorithm could also identify the tumor area better, the area selection range was too large, so that it was easy to include the background area, showing poor segmentation effect.

**Figure 3:**
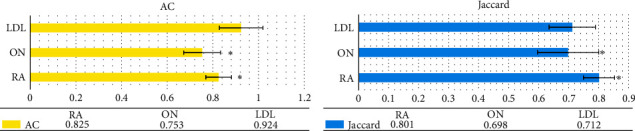
Comparison on AC and Jaccard of various algorithms. (a) The AC indicator and (b) the Jaccard indicator. ^*∗*^ indicates that the difference was statistically obvious in contrast to the LDL algorithm (*P* < 0.05).

**Figure 4:**
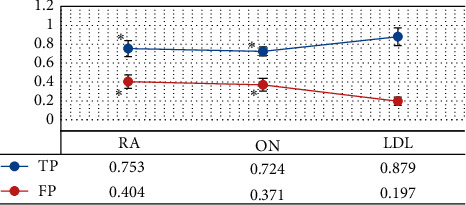
Comparison on TP and FP of various algorithms. ^*∗*^ suggests that the difference was statistically obvious in contrast to the LDL algorithm (*P* < 0.05).

The quantitative indicators were compared to further clarify the segmentation performances of the three algorithms. As shown in Figures 3 and 4, the AC, Jaccard, and TP values of the LDL algorithm were greatly larger than those of the RA and ON algorithms, showing statistically obvious differences (*P* < 0.05); the FP value of the LDL algorithm was much smaller in contrast to that of the RA and ON algorithms, showing statistically great difference (*P* < 0.05).

### 3.2. Comparison on Basic Information of the Patients

**Figure 5:**
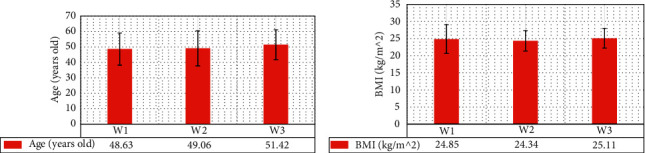
Comparison on age and BMI of patients in three groups. (a, b) The statistical results of age and BMI, respectively.

**Figure 6:**
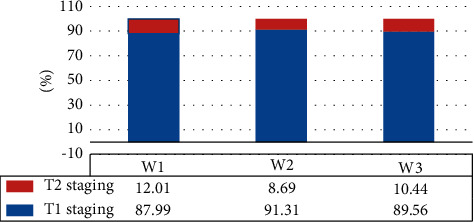
Comparison on tumor staging proportions of patients in three groups.

As illustrated in Figures 5 and 6, the pairwise comparisons of age, body mass index (BMI), and tumor stage ratio of patients in W1, W2, and W3 groups were not statistically observable (*P* > 0.05).

**Figure 7:**
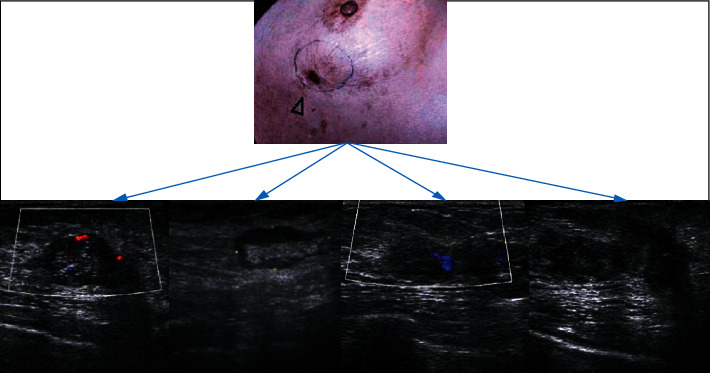
The ultrasound images of a 46-year-old female patient with breast cancer. The patient went to hospital for examination due to a lump in the left breast with obvious pain.

Figure 7 shows an ultrasound image of a 46-year-old female patient with breast cancer. Ultrasound showed that the breast tissues on both sides were slightly thickened, the lobule structure was slightly thick and disordered, and the internal distribution was uneven. There were two hypoechoic masses of different sizes in the upper outer quadrant, with irregular shapes, and there was no echo in the capsule. The internal echo was uneven, and several sand particles showed strong echoes with weak sound shadows. In addition, the color Doppler ultrasound showed that there was no blood flow signal, right axillary lymph node was not reached, and multiple hypoechoic nodules were visible in the axillary.

### 3.3. Comparison on Resection Volume of Tumor

**Figure 8:**
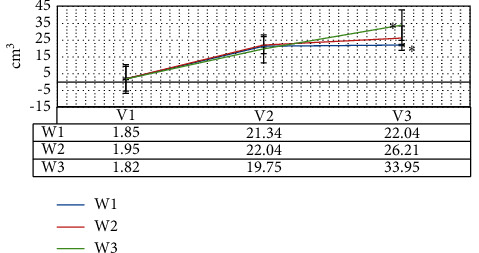
Comparison on resection volume of tumor. ^*∗*^ indicates that the statistically visible difference could be found in contrast to the W1 group (*P* < 0.05).

As given in Figure 8, the tumor volume and ideal resection volume of patients in the W1, W2, and W3 groups were relatively close, and the difference was not statistically significant (*P* > 0.05); the actual resection volume in the W1 group was the closest to the ideal resection volume and was greatly smaller than that in the W2 and W3 groups (*P* < 0.05).

**Figure 9:**
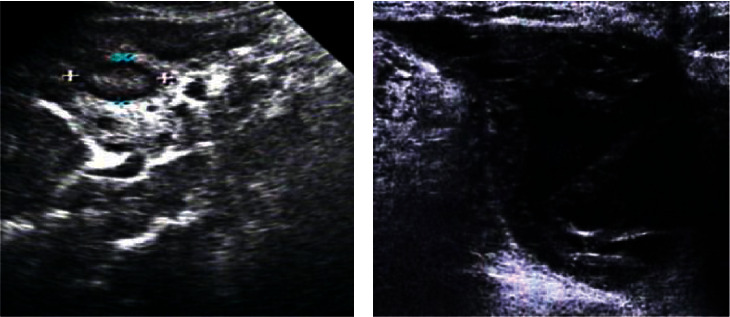
Images of ultrasound reexamination of some patients after surgery. (a) Reexamination result of a 56-year-old female patient. (b) Reexamination result of a 43-year-old female patient.

Figure 9 shows the images of ultrasound reexamination in some patients after surgery. As shown in Figure 9(a), the ultrasound reexamination found that the right breast cancer of the patient was treated for one year after radiotherapy; the sonographic features were obvious, and the multiple hypoechoic nodules showed target ring signs, which were multiple liver metastases. Figure 9(b) reveals that the patient's left side breast cancer had an obstruction of lymphatic drainage, that is, lymphatic fluid accumulated in the axilla and formed a lymphatic cyst.

### 3.4. Comparison of Patient Margin Performance

**Figure 10:**
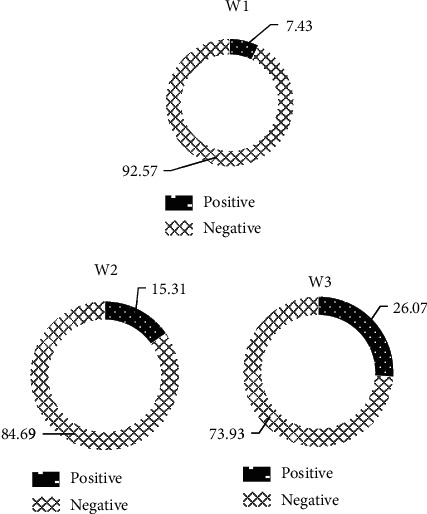
Comparison on positive margins of patients in three groups.

As revealed in Figure 10, the positive margins and negative margins of the patients in the W1 group were 7.43% and 92.57%, respectively; the positive and negative margins of the patients in the W2 group were 15.31% and 84.69%, respectively, and the positive and negative margins of the patients in the W3 group were 26.07% and 73.93%, respectively. It was clear that the positive margins of the patients in the W1 group were dramatically less than those in the W2 and W3 groups, and the differences were statistically observable (*P* < 0.05).

**Figure 11:**
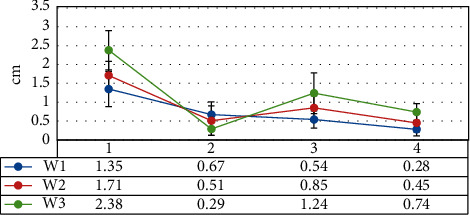
The longest and shortest margins of the three groups of patients.1 means the longest margin, 2 refers to the shortest margin, 3 marks the difference between the longest margin and the ideal margin, and 4 represents the difference between the shortest margin and the ideal margin value.

Figure 11reveals that the longest margin (1.35 cm) of the patients in the W1 group was smaller in contrast to that of the patients in the W2 and W3 groups, while the shortest margin (0.67 cm) was greater. Based on the difference between the longest resection margin and the ideal resection margin and the difference between the shortest resection margin and the ideal resection margin, it can be concluded that the longest and shortest resection margins of the patients in group W1 were closer to the ideal resection margin, and the variation among individuals was smaller.

### 3.5. Analysis on Postoperative Cosmetic Effect of Patients

**Figure 12:**
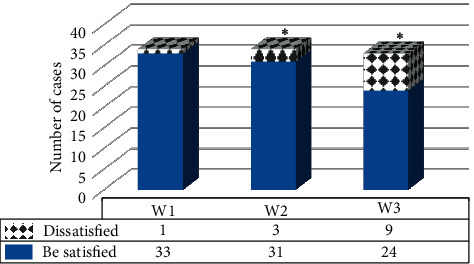
Comparison on postoperative cosmetic effects of patients in different groups. ^*∗*^ indicates that the statistically visible difference could be found in contrast to the W1 group (*P* < 0.05).

The postoperative cosmetic effects of the three groups of patients were compared, and the results are shown in Figure 12. There was 1 patient in the W1 group who was not satisfied with the cosmetic effect because there was an obvious shift in the position of the nipple. In the W2 group, 3 patients were not satisfied with the cosmetic effect: 1 case suffered from a significant deviation in the position of the nipple, and 2 suffered from larger breast scar areas. In the W3 group, 9 patients were not satisfied with the cosmetic effect, including 3 patients with obvious deviation of the nipple position, 2 patients with large breast scar areas, 3 patients with visible breast deformation, and 1 patient with postoperative infection. As a result, patients in the W1 group were more satisfied with the cosmetic effect than those in the W2 and W3 groups, and the differences were statistically notable (*P* < 0.05).

## 4. Discussion

With the development of medical technology, the clinical treatment of breast cancer is constantly updated and progressed. Great changes have been realized from the initial huge traumatic treatment to today's minimally invasive treatment. The breast-conserving surgery has become the best treatment way for early breast cancer in current years. On the other hand, the application of imaging methods in the surgical process is the current trend. As a diagnostic tool for early breast cancer, ultrasound has also been used in breast cancer, which shows very important value [15]. Therefore, a deep LDL model was designed for breast cancer patients during the breast-conserving surgery in this study. The semiautomatic segmentation algorithm based on the region growing and active contour technology (RA) and the segmentation model based on optimized nearest neighbors (ON) were introduced for comparison to further analyze the performance of the designed model. The results revealed that the tumor area segmented by the LDL algorithm was closer to the real tumor area; although the RA algorithm and the ON algorithm could also identify the tumor area better, the area selection range was too large, so that it was easy to include the background area, showing poor segmentation effect. The quantitative results suggested that the AC, Jaccard, and TP values of the LDL algorithm were greatly larger than those of the RA and ON algorithms, and the FP value of the LDL algorithm was much smaller in contrast to that of the RA and ON algorithms, showing statistically differences (*P* < 0.05). Such results were similar to the findings of Wang et al. [16], indicating that the LDL algorithm constructed in this study showed a good effect on tumor segmentation, improving the accuracy of tumor segmentation, so it was suitable for clinical promotion.

According to the difference in intraoperative guidance methods, 102 female patients with early breast cancer were divided into three groups: 34 cases in W1 group (ultrasound guidance based on deep learning segmentation model), 34 cases in W2 group (ultrasound guidance), and 34 cases in W3 group (palpation guidance). The results suggested that the actual resection volume of the patients in the W1 group was the closest to the ideal resection volume, which was much smaller in contrast to that of the patients in the W2 and W3 groups, showing statistical differences (*P* < 0.05). The amount of breast tissue removed will affect the appearance of the patient's breast after surgery. Excessive removal may cause the breast to collapse and deform, so it is necessary to reduce the amount of breast tissue resection under the premise of negative margins during the surgery to improve the success rate of breast preservation [17]. It was concluded that the ultrasound images based on the deep LDL model could effectively improve the AC of tumor resection and reduce the probability of normal tissue being resected. The positive margins of the patients in the W1 group were statistically lower than those in the W2 and W3 groups (*P* < 0.05), which was different with the research results of Guo et al. [18]. Resection margins are closely related to the postoperative recurrence of patients. Obtaining satisfactory negative margins is a topic of clinical concern. It was found in this study that ultrasound images based on the deep LDL model could effectively reduce the positive rate of resection margins in patients.

There are many side effects after breast cancer surgery, and the breast cosmetic effect has a great impact on the patient's physical and mental health. Previous studies have shown that breast asymmetry will make women's quality of life worse [19]. A follow-up survey was performed for the three groups of patients 6 months after the surgery. There was 1 patient in the W1 group who was not satisfied with the cosmetic effect because there was an obvious shift in the position of the nipple. In the W2 group, 3 patients were not satisfied with the cosmetic effect: 1 case suffered from a significant deviation in the position of the nipple, and 2 suffered from larger breast scar areas. In the W3 group, 9 patients were not satisfied with the cosmetic effect, including 3 patients with obvious deviation of the nipple position, 2 patients with large breast scar areas, 3 patients with visible breast deformation, and 1 patient with postoperative infection. Such results revealed that the ultrasound image based on the deep LDL model exerted reliable positive impacts on the cosmetic effect of the patient's breast after surgery, which was different from the results obtained by An et al. [20]. The possible reason may be that the resection amount of breast tissue during surgery could affect the postoperative cosmetic effect, resulting in significant differences in the cosmetic effect of the three groups.

## 5. Conclusion

A deep LDL model was designed, and the semiautomatic segmentation algorithm RA and the segmentation model ON were introduced for comparison. The designed algorithm was applied to the breast-conserving surgery of breast cancer patients. According to the difference of intraoperative guidance methods, 102 female patients with early breast cancer were divided into three groups: 34 cases in W1 group (ultrasound guidance based on deep learning segmentation model), 34 cases in W2 group (ultrasound guidance), and 34 cases in W3 group (palpation guidance). It was found that ultrasound images based on the deep LDL model effectively improved the tumor resection AC and negative margins, reduced the probability of normal tissue being removed, and enhanced the postoperative breast cosmetic effect. However, the constructed deep LDL model cannot be applied to all situations due to the complicated tumor edge characteristics, and the number of patients was small, so further empirical research was necessary in the follow-up. In summary, the image segmentation model proposed in this study showed a good application value for the implementation of clinical breast-conserving surgery for breast cancer.

## Data Availability

The data used to support the findings of this study are available from the corresponding author upon request.
